# Quantitative and qualitative assessment of a pituitary neuroendocrine tumor’s T2-signal intensity in acromegaly – a call for unification

**DOI:** 10.3389/fendo.2024.1441745

**Published:** 2024-11-20

**Authors:** Magdalena Godlewska-Nowak, Anna Grochowska, Grzegorz Zieliński, Anna Bogusławska, Dariusz Adamek, Maria Maksymowicz, Alicja Hubalewska-Dydejczyk, Aleksandra Gilis-Januszewska

**Affiliations:** ^1^ Chair and Department of Endocrinology, Jagiellonian University Medical College, Krakow, Poland; ^2^ Doctoral School of Medical and Health Sciences, Jagiellonian University, Krakow, Poland; ^3^ Department of Radiology, Jagiellonian University Medical College, Krakow, Poland; ^4^ Department of Neurosurgery, Military Institute of Medicine, Warsaw, Poland; ^5^ Department of Pathomorphology, Jagiellonian University Medical College, Krakow, Poland; ^6^ Department of Cancer Pathomorphology, Maria Sklodowska-Curie National Research Institute of Oncology, Warsaw, Poland

**Keywords:** acromegaly, pituitary neuroendocrine tumor, T2-signal intensity, magnetic resonance, somatostatin analogue

## Abstract

**Introduction:**

The T2-signal intensity (SI) of somatotroph pituitary neuroendocrine tumors (sPitNET) is associated with treatment response and granulation pattern. Our aim was to evaluate SI assessment methods and their clinical implications, including responsiveness to preoperative first-generation somatostatin analogs (SSA).

**Methods:**

This single-center, observational study included unselected, consecutive patients with newly diagnosed acromegaly. Out of 109 treatment-naïve patients, 69 were eligible. The qualitative Visual Method involved a visual comparison of the sPitNET with the temporal gray matter. The Three Tissue Method compared the quantified SI of the sPitNET, temporal white matter, and gray matter. The signal intensity ratio of the sPitNET vs. gray matter (GM-SIR) was calculated. Tumors were divided into three groups: hyperintense (HYPER), isointense (ISO), and hypointense (HYPO) according to the Visual Method, Three Tissue Method, and GM-SIR. These groups were compared in terms of demographic, radiological, and biochemical features. The SI assessment methods were investigated for their ability to predict preoperative SSA responsiveness.

**Results:**

SI assessment methods classified SI type correspondingly in 58-75.4% of cases. ISO constituted 39-49% of the analyzed sPitNETs. All methods identified significant differences in tumor volume between the SI groups, with HYPO being more biochemically active per tumor volume unit. According to the Three Tissue Method, patients with ISO had the youngest age at diagnosis and onset. According to the Visual Method, ISO had a lower chance of achieving insulin-like growth factor 1 (IGF1) normalization compared to HYPO (odds ratio (OR) 0.089, confidence interval (CI) 0.015-0.538, p= 0.008)), with no differences between HYPER and HYPO. Only the Visual Method predicted the IGF1 normalization after SSA. HYPER and ISO sPitNETs were classified in electron microscopy as both densely and sparsely granulated. Bihormonal tumors presented only as HYPO and ISO. According to the Three Tissue Method, no HYPO was diagnosed with sparse granulation.

**Discussion:**

We demonstrated discrepancies between the SI assessment methods. The Visual Method predicted the outcome of preoperative treatment with SSA. Clinically, ISO behaved similarly to HYPER. Further studies are needed to unify SI assessment and improve its clinical applicability in acromegaly.

## 1 Introduction

Pituitary magnetic resonance imaging (MRI) remains the gold standard in diagnosing the somatotroph pituitary neuroendocrine tumors (sPitNET). Many patients with acromegaly require pharmacological treatment, either following surgical failure or when surgery is not feasible or accepted by the patient (1–3). The MRI-based T2-weighted signal intensity (SI) of the sPitNET has been investigated as a possible non-invasive marker of the tumor’s clinical behavior and response to pharmacotherapy. sPitNETs are heterogenous in terms of SI, manifesting as hyperintense (HYPER), isointense (ISO), or hypointense (HYPO) sellar lesions. In a study including 174 PitNETs, hypointensity was exclusively associated with dense granulation of somatotropinomas (4). Hypointensity has also been associated with responsiveness to the first-generation of somatostatin analogs (SSAs), both preoperatively (5–7) and after surgical failure (8). Tumors with a higher SI have been associated with a sparse granulation pattern, they are frequently unresponsive to SSAs but have a good clinical response when treated with pasireotide (4, 6, 9, 10). Recent guidelines underline the usefulness of SI in the management of acromegaly (1, 2, 11). However, there is no unified tool to assess SI. A comparison of the published studies reveals certain discrepancies between their methodologies and results (**Table 1**). SI has been approached qualitatively and quantitatively, as summarized by Bonneville et al. (12). Qualitative assessment, based on the visual comparison of the sPitNET with a reference tissue (Visual Method) is commonly used (4, 5, 12–14). Quantitative assessment involves delineating the region of interest (ROI) in the solid part of the sPitNET and the reference tissues. The SI is then quantified within the ROI and can be expressed as a ratio: the sPitNET’s SI is divided by the SI of the reference tissue. Gray matter (6, 7, 12), white matter (15, 16), or cerebrospinal fluid (16) have been used as reference structures. Alternatively, the sPitNET’s quantified SI values can be compared to the quantified SI values of gray matter and white matter and used to classify sPitNETs into different intensity groups. A higher sPitNET SI than that of the gray matter corresponds to hyperintensity. A lower sPitNET’s SI value than that of the gray matter but higher than the SI of the white matter defines isointensity. Finally, a lower sPitNET SI than that of the white matter indicates hypointensity (Three Tissue Method) (12).

**Table 1:** Various signal intensity assessment methods proposed in the current literature.

Article	MRI sequences used for assessment	Reference Tissues	Assessment method	Frequency of Signal Intensity group (%)		
				HYPER	ISO	HYPO
Hagiwara et al., 2003 (4)	T2	White and gray matter	Visual	28%	32%	40%
Heck et al., 2012 (5)	T2	White and gray matter of the temporal lobe	Visual, Three Tissue Method when visual assessment not possible	40%	33%	27%
Heck et al., 2015 (6)	T2	Gray matter	Visual	21%	42%	37%
			Normalized Histogram	Not available		
Potorac et al., 2015 (14)	T2	Healthy pituitary, when not visible: gray matter	Visual	26%	21%	53%
			Quantitative	Verification of 29 cases, 93% compatible results		
Potorac et al., 2016 (7)	T2	Healthy pituitary, gray matter	Visual	16%	14%	70%
			Relative Signal Intensity (GM-SIR)	Not available		
Shen et al., 2016 (15)	T2, T1	White matter of the frontal lobe	Relative Signal Intensity (WM-SIR)	Not available		
Alhambra-Expósito et al., 2018 (13)	T2	Healthy pituitary, when not visible: gray matter	Visual	59%	41 %	0%
Dogansen et al., 2018 (10)	T2	Healthy pituitary, when not visible: gray matter	Visual	26%	21%	53%
Bonneville et al., 2019 (12)	T2	White and gray matter of the temporal lobe	Visual	5%	36%	59%
			Three Tissue Method	33%	47%	20%
			Relative Signal Intensity (GM-SIR)	12%	52%	36%
Lewis et al., 2022 (16)	T2, T1	White matter, cerebro-spinal fluid	Visual, Relative Signal Intensity (including WM-SIR)	Not available		

HYPER, hyperintense somatotroph pituitary neuroendocrine tumor; ISO, isointense somatotroph pituitary neuroendocrine tumor; HYPO, hypointense somatotroph pituitary neuroendocrine tumor; GM-SIR, gray matter signal intensity ratio; WM-SIR, white matter signal intensity ratio.

Frequency of hyperintense, isointense, and hypointense somatotroph pituitary neuroendocrine tumors according to each method (if available).

## 2 Materials and methods

### 2.1 Study design and objective

**Figure 1:**
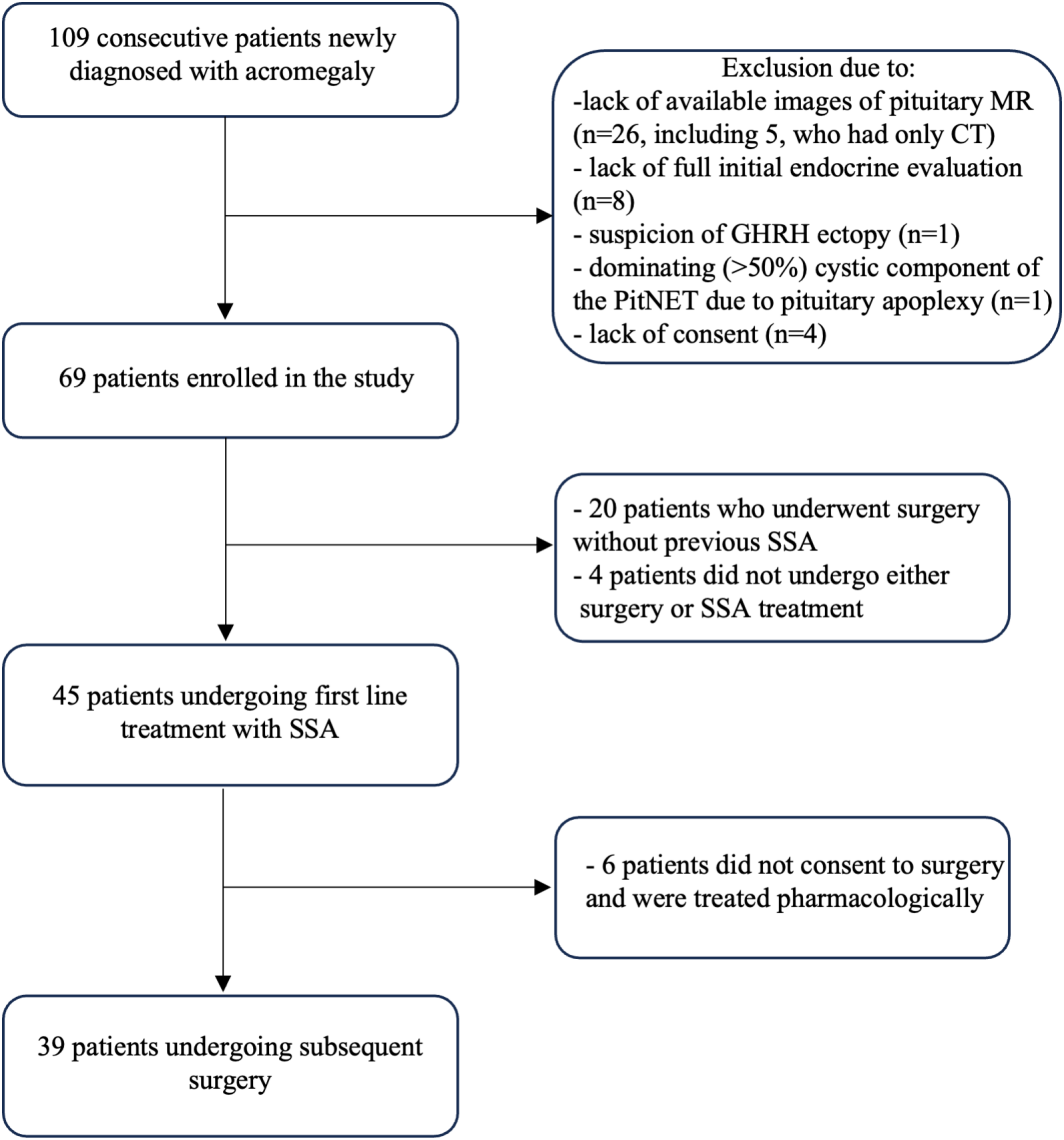
Methodology of the study: flow chart.

This non-interventional single-center observational study was conducted at the Chair and Department of Endocrinology, Jagiellonian University Medical College in Krakow. We identified 109 consecutive, unselected patients newly diagnosed with acromegaly between 2012 and 2022. The inclusion and exclusion criteria for the recruitment of patients are presented in **Figure 1**. Finally, 69 patients were included in the analysis. The study was conducted according to the guidelines of the Declaration of Helsinki and approved by the Ethics Committee of the Jagiellonian University (1072.6120.72.2020). It is a part of Jagiellonian University statutory research (N41/DBS/000407). Patients gave written informed consent.

Our objective was to evaluate qualitative and quantitative methods of SI assessment and their clinical implications for sPitNETs including responsiveness to preoperative SSA.

### 2.2 Radiological parameters

Pituitary images were obtained using at least 1.5 Tesla MR scanners and contained coronal T2-weighted sequences, with a slice thickness of 3 mm. For the purpose of SI and tumor volume (TV) measurement, syngo.via (Siemens) was used. Assessment was performed in all cases by a single researcher (MG) and verified by a radiologist experienced in pituitary MRI interpretation (AG). MR images were also reviewed by an expert pituitary neurosurgeon (GZ). The SI assessment methods proposed by Bonneville et al. (12) were adapted:

1. Qualitative assessment of the SI (Visual Method). The intensity of the solid part of the sPitNET was visually compared to the intensity of the gray matter of the adjacent temporal lobe. sPitNET was classified as HYPER when its intensity appeared higher than that of the gray matter; as ISO, when its intensity was similar to the intensity of the gray matter, and as HYPO, when its intensity appeared lower than the intensity of the gray matter.
2. Quantitative assessment of the SI: the ROIs were designated in the solid part of the sPitNET (12), in the white matter, and in the gray matter of the temporal lobe. SI was measured in each ROI and expressed as a mean value of SI in this area to exclude the influence of the possible heterogeneity (5). Two quantitative methods of classification (12) were used:

a) The signal intensity ratio of the sPitNET vs. gray matter (GM-SIR) was calculated by dividing the PitNET’s mean SI by the mean SI of the temporal gray matter. A GM-SIR ≥ 1.2 classified the PitNET as HYPER and a GM-SIR > 0.8 but <1.2 as ISO. A GM-SIR ≤ 0.8 classified the tumor as HYPO.
b) The Three Tissue Method was based on the comparison of the quantified SI of the sPitNET, gray matter, and white matter. Mean sPitNET’s SI higher than the mean SI of gray matter classified the tumor as HYPER. SI between the SI value of gray matter and white matter classified the sPitNET as ISO. sPitNET’s SI lower than white matter’s SI classified the tumor as HYPO.

Other analyzed radiological parameters included tumor maximal diameter (cm), TV measured by manual delineation of the volume of interest within the tumor tissue (cm^3^), invasion of the cavernous sinuses expressed using the Knosp scale, and the presence of optic chiasm compression by the sPitNET.

### 2.3 Baseline hormonal evaluation and response to SSA

Biochemical confirmation of acromegaly was based on insulin-like growth factor 1 (IGF1) concentration above the normal range for age and sex, with a lack of growth hormone (GH) suppression (<1 μg/l) after oral glucose load (75 g). IGF1 was expressed as the ratio of IGF1 concentration and its upper limit of age- and sex-adjusted normal range (IGF1/ULN). The methodology of GH and IGF1 assessment has been described elsewhere (17). GH concentrations were presented as fasting and nadir concentrations after oral glucose load. GH was also expressed as GH concentration and TV (cm^3^) ratio (GH/TV [μg/l *cm^3^]). Prolactin (PRL) concentration was expressed as the ratio of PRL concentration and its upper limit of normal range (PRL/ULN). In total, 45 patients were preoperatively treated with lanreotide autogel, 120 mg every 4 weeks, and 30 mg octreotide LAR every 4 weeks, for 3-6 months. The pharmacotherapy was implemented as recommended by the Polish guidelines (3). Hormonal evaluation, including GH concentration and IGF1/ULN assessment, was performed in all cases after 3 to 6 months of treatment. Full biochemical control during pharmacological treatment was defined as achieving IGF1/ULN <1 and GH concentration < 2.5 μg/l (12, 18). Separate alternative criteria of response were the normalization of IGF1 alone (IGF1/ULN <1) and isolated control of GH concentration < 2.5 μg/l. Percentage reduction of IGF1 and GH after SSA treatment was calculated. All patients were offered surgical treatment and ten patients did not consent to surgery.

### 2.4 Histopathology

In total, 59 of the enrolled patients underwent surgery and 41 had available histopathological results. Of the latter, 31 results, as well as 28 electron microscope analyses, came from patients who were presurgically treated with SSA. Tumors were immunophenotyped with antibodies against all tropic anterior pituitary hormones, the alpha subunit of glycoprotein hormone (αSU), and PitNET lineage-specific transcription factors (TPIT, PIT-1, SF-1). Ki-67 expression was analyzed. sPitNETs were divided into sparsely granulated (SG), densely granulated (DG), or bihormonal GH-PRL tumors (mammosomatotroph tumors and mixed tumors of densely granulated somatotroph and lactotroph cells) based on the electron microscopy.

### 2.5 Statistical analysis

Statistical analysis was performed using IBM SPSS Statistics, version 29. The significance level was set at 0.05 unless stated otherwise. Categorical variables were expressed as frequencies and evaluated with Pearson’s chi-square or Fisher’s exact tests (unanimity of SI assessment methods). For 3 x 2 contingency tables, *post hoc* analysis for categorical data was assessed in 2 × 2 contingency tables. An adjusted p value of < 0.0083 was considered significant in the *post hoc* multiple comparisons (frequency of females, optic chiasm compression, and IGF1 normalization). The Shapiro–Wilk test was applied to check the distribution of continuous variables. Quantitative variables were presented as mean +/- SD or median with interquartile range (Q1; Q3). Analysis of variance (ANOVA) with Bonferroni *post hoc* analysis (age at diagnosis, age at onset, IGF1 reduction after SSA) and the Kruskal–Wallis test, with pairwise comparisons (TV, fasting and nadir GH concentrations, GH/TV, IGF1/ULN, PRL/ULN, IGF1, and GH reduction after SSA), were used to compare continuous variables between HYPER, ISO, and HYPO. Pearson’s or Spearman’s correlations were applied to establish associations between GM-SIR and biochemical, radiological, and demographic variables. Univariate logistic regression was applied to investigate the influence of variables on pharmacotherapy outcomes.

## 3 Results

### 3.1 Patients’ characteristics

Among the 69 patients included, 53.6% were females. The age at diagnosis (mean, +/- SD) was 45.3 +/- 14.4 years, while the age at the onset of symptoms (mean, +/- SD) was 38.4 +/- 14.1 years. The diagnostic delay (median, Q1;Q3) was 5 years (2.5;10). The GH fasting concentration (median, Q1;Q3) was 8.6 μg/l (4.5; 15.2), while the nadir GH level after glucose load (median, Q1;Q3) was 8 μg/l (3.9; 15.9). Among the included patients, IGF1/ULN (median, Q1;Q3) was 2.0 (1.7;2.5). The largest diameter sPitNET (median, Q1;Q3) was 14 mm (11; 22). Based on sPitNET diameter, 13 patients were diagnosed with microadenomas, 54 with macroadenomas, and in 2 cases giant sPitNETs were diagnosed. Furthermore, 25 patients (36.2%) harbored sPitNETs that were homogenous in their signal intensity and 5 patients had features of having undergone a pituitary tumor apoplexy.

### 3.2 Signal intensity classification according to various methods

The results of the sPitNET classifications are depicted in **Table 2**. The Visual Method and GM-SIR classified sPitNETs correspondingly in 75.4% of the cases, the Visual Method and the Three Tissue Method in 58%, and GM-SIR and the Three Tissue Method in 65.2% of cases. According to all methods, no tumors classified as HYPER by one method were categorized as HYPO by another SI assessment method, and vice versa. Discrepancies in category assignments were observed between HYPO and ISO, as well as between HYPER and ISO, as detailed in **Supplementary Table 1**. The differences in frequencies of patients assigned to each SI category were significant for all comparisons of the methods (p<0.001).

**Table 2:** Signal Intensity classification of the Somatotroph Pituitary Neuroendocrine Tumors according to various methods of Signal Intensity assessment.

Signal Intensity assessment method	Frequency of the sPitNET type according to signal intensity (n; %)		
	HYPER	ISO	HYPO
**Visual Method**	18; 26.1%	27; 39.1%	24; 34.8%
**GM-SIR**	13; 18.8%	34; 49.3%	22; 31.9%
**Three Tissue Method**	28; 40.6%	28; 40.6%	13; 18.8%

HYPER, hyperintense; ISO, isointense; HYPO, hypointense somatotroph Pituitary Neuroendocrine Tumor; GM-SIR, gray matter signal intensity ratio.

### 3.3 Demographic parameters

Demographical parameters are presented in **Table 3**. Females predominated in HYPER and HYPO, but not in ISO, regardless of the SI assessment method. Only in the GM-SIR-based division did the differences reach statistical significance: up to 84.6% of patients with HYPER were females. According to the Three Tissue Method, we discovered statistically significant differences between the three SI groups in terms of age at diagnosis as well as age at the onset: patients with ISO, with a median age at diagnosis of 41 years and a median age at the onset of 34 years, were statistically younger than patients with HYPO.

**Table 3:** Demographic parameters in hyperintense, isointense, and hypointense somatotroph pituitary neuroendocrine tumors.

	HYPER	ISO	HYPO	p value	*Post-hoc* analyses between groups - p value		
					HYPER vs. ISO	HYPER vs. HYPO	ISO vs. HYPO
Visual Method							
Age at diagnosis, years (mean; SD)	47.4; 18.9	42.7; 11.6	47.4; 13.2	0.649	-	-	-
Age at onset, years (mean, SD)	38.7; 18.5	37.3; 11.1	40.8; 13.9	0.387	–	–	–
Sex, females (n; %)	10; 55.6%	12; 46.2%	15; 60%	0.601	-	-	-
GM-SIR							
Age at diagnosis, years (mean, SD)	48.9; 18	40.9; 12.9	52; 13	0.065	-	-	-
Age at onset, years (mean, SD)	38.9; 17.2	35.6; 12.89	44.9; 14.4	0.065	-	-	-
Sex, females (n; %)	11; 84.6%	14; 41.2%	12; 54.5%	**0.028**	**0.0075** ^a^	0.07^a^	0.33^a^
Three Tissue Method							
Age at diagnosis, years (mean, SD)	46.5; 16.7	40.7; 9.9	56.7; 13.8	**0.033**	0.69	0.258	**0.028**
Age at onset, years (mean, SD)	39.6; 16	34.3; 9.2	49.6; 16.8	**0.007**	0.594	0.099	**0.005**
Sex, females (n; %)	19; 67.9%	11; 39.3%	7; 53.8%	0.1	-	-	-

Bold values are statistically significant (p < 0.05).

a Bonferroni adjustment of p value necessary for multiple comparisons: statistical significance for p<0.0083.

HYPER, hyperintense somatotroph pituitary neuroendocrine tumor; ISO, isointense somatotroph pituitary neuroendocrine tumor; HYPO, hypointense somatotroph pituitary neuroendocrine tumor; GM-SIR, gray matter signal intensity ratio.

### 3.4 Radiological parameters

The radiological characteristics of SI groups are presented in **Table 4**. According to the Visual Method, HYPER had statistically higher TV than HYPO. According to GM-SIR, HYPER was also significantly larger than HYPO. Assessment with the Three Tissue Method revealed that ISO had significantly higher TV than HYPO. None of the methods showed statistically significant differences between HYPER and ISO. Nor were there any differences between HYPER, ISO, and HYPO in terms of the tumor’s largest diameter, regardless of the SI assessment method. According to GM-SIR, optic chiasm compression was more frequent in HYPER than in HYPO. HYPER compressed the optic chiasm twice as frequently as ISO, however, this difference did not reach statistical significance. The Visual Method and the Three Tissue Method did not show statistical differences between groups in terms of the frequency of optic chiasm compression. The frequency of the cavernous sinus invasion did not differ between SI groups, regardless of the classification method.

**Table 4:** Radiological parameters at baseline in hyperintense, isointense and hypointense somatotroph Pituitary Neuroendocrine Tumors.

	HYPER	ISO	HYPO	p value	*Post-hoc* analyses between groups- p value		
					HYPER vs. ISO	HYPER vs. HYPO	ISO vs. HYPO
Visual Method							
Maximal tumor diameter, median, Q1; Q3 (mm)	18.5 (13; 24)	13 (11; 26.25)	13 (9; 16.25)	0.123	-	**-**	-
Tumor volume, median, Q1; Q3 (cm^3^)	2.86 (1.11; 10.21)	1.2 (0.71; 2.46)	0.77 (0.51; 2.24)	**0.017**	0.107	**0.016**	1.0
Cavernous sinus invasion (n; %)	11; 61%	11; 42.3%	12; 48%	0.465	-	-	-
Optic chiasm compression (n; %)	7; 38.9%	4; 15.4%	5; 20%	0.172	-	-	-
GM-SIR							
Maximal tumor diameter, median, Q1; Q3 (mm)	17 (12; 27)	14 (10; 16)	12.5 (10.25; 22)	0.081	**-**	-	**-**
Tumor volume, median, Q1; Q3 (cm^3^)	2.21 (1.0; 5.79)	1.83 (0.8; 9.55)	0.74 (0.4; 1.54)	**0.006**	1.0	**0.013**	**0.025**
Cavernous sinus invasion (n; %)	9; 69.2%	16; 47.1%	9; 40.9%	0.252	**-**	-	-
Optic chiasm compression (n; %)	6; 46.2%	8; 23.5%	2; 9.1%	**0.043**	0.129^a^	**0.032^a^**	0.285^a^
Three Tissue Method							
Maximal tumor diameter, median, Q1; Q3 (mm)	14.5 (10;20.5)	15 (13; 26.75)	12 (8.5; 16.5)	0.073	-	-	**-**
Tumor volume, median, Q1; Q3 (cm^3^)	1.29 (0.73-5.03)	1.83 (1.07-7.51)	0.58 (0.43-1.15)	**0.005**	0.853	0.056	**0.04**
Cavernous sinus invasion (n; %)	13; 46.4%	15; 53,6%	6; 46.2%	0.84	-	-	-
Optic chiasm compression (n; %)	8; 29.6%	7; 25%	1; 7.7%	0.323	-	-	-

Bold values are statistically significant (p < 0.05).

a Bonferroni adjustment of p value necessary for multiple comparisons: statistical significance for p<0.0083.

HYPER, hyperintense; ISO, isointense; HYPO, hypointense somatotroph Pituitary Neuroendocrine Tumor; GM-SIR, gray matter signal intensity ratio.

### 3.5 Baseline biochemical parameters and response to SSA

Baseline characteristics are shown in **Table 5**. None of the methods (Visual Method, GM-SIR, Three Tissue Method) showed statistically significant differences between SI groups in GH fasting or nadir (data not shown in **Table 5**) concentrations as well as IGF1/ULN. According to the Visual Method, HYPO had a higher GH/TV than HYPER. When the SI assessment based on the GM-SIR was used, a similar tendency was discovered, but it did not reach statistical significance in the *post hoc* comparisons. Classification according to the Three Tissue Method showed that both HYPER and ISO had significantly lower GH/TV than HYPO. No method revealed differences between HYPER and ISO in GH/TV. Median PRL/ULN did not differ between SI groups, according to all methods (data not shown in **Table 4**). After SSA treatment, we found no differences between SI groups (according to all SI assessment methods) in GH or IGF1 percentage reduction as well as in the frequency of achieving isolated GH control or full biochemical control. Details of the biochemical response to SSA are depicted in **Table 6**. Only the division according to the Visual Method revealed differences in the frequency of achieving IGF1 normalization after SSA. However, *post hoc*, no significant differences were discovered between HYPER, ISO, and HYPO.

**Table 5:** Baseline biochemical parameters in hyperintense, isointense, and hypointense somatotroph pituitary neuroendocrine tumors.

	HYPER	ISO	HYPO	p value	*Post-hoc* analyses between groups- p value		
					HYPER vs. ISO	HYPER vs. HYPO	ISO vs. HYPO
Visual Method							
Fasting GH, median, Q1; Q3 (μg/l)	9.5 (4.0; 17.2)	8.3 (3.5; 22.4)	8.6 (6.2; 12.1)	0.717	-	-	-
GH/TV, median, Q1; Q3 (μg/l *cm^3^)	3.1 (1.1; 5.2)	6.56 (2.5; 11.5)	10.2 (3.0; 17.9)	**0.048**	0.276	**0.044**	1.0
IGF1/ULN, median, Q1; Q3	2.06 (1.59; 2.94)	1.93 (1.69; 2.58)	2.11 (1.66; 2.73)	0.925	-	-	-
GM-SIR							
Fasting GH, median, Q1; Q3 (μg/l)	10.5 (3.9; 28.7)	9.33 (4.4; 17.6)	8.37 (5.5; 9.7)	0.566	-	-	-
GH/TV, median, Q1; Q3 (μg/l *cm^3^)	4.5 (1.1; 12.1)	3.4 (1.9; 8.3)	10.5 (4.1; 18.4)	**0.048**	1.0	0.054	0.245
IGF1/ULN, median, Q1; Q3	1.96 (1.58; 2.61)	1.99 (1.68; 2.93)	2.08 (1.51; 2.68)	0.908	-	-	-
Three Tissue Method							
Fasting GH, median, Q1; Q3 (μg/l)	8.9 (3.8; 14.6)	8.4 (4.8; 15.7)	8.6 (6.2; 11.4)	0.979	-	-	**-**
GH/TV, median, Q1; Q3 (μg/l*cm^3^)	5.4 (1.7; 12.7)	3.4 (2.3; 6.6)	14 (8.5-22.3)	**0.006**	1.0	**0.04**	**0.004**
IGF1/ULN, median, Q1; Q3	1.89 (1.6-2.7)	2.06 (1.7-2.6)	2.21 (1.4-3.1)	0.928	-	-	**-**

Bold values are statistically significant (p < 0.05).

HYPER, hyperintense somatotroph pituitary neuroendocrine tumor; ISO, isointense somatotroph pituitary neuroendocrine tumor; HYPO, hypointense somatotroph pituitary neuroendocrine tumor; GM-SIR, gray matter signal intensity ratio; GH, growth hormone; GH/TV, growth hormone concentration and tumor volume ratio; IGF1/ULN, IGF1 concentration and its upper limit of normal ratio adjusted for age and sex.

**Table 6:** Biochemical parameters after preoperative treatment with somatostatin analogues in hyperintense, isointense and hypointense somatotroph Pituitary Neuroendocrine Tumors.

	HYPER	ISO	HYPO	p value	*Post-hoc* analyses between groups- p value		
					HYPER vs. ISO	HYPER vs. HYPO	ISO vs. HYPO
Visual Method							
GH reduction, median, Q1; Q3 (%)	71.5 (11; 82.9)	60 (13; 87.6)	69.4 (17.2; 91.5)	0.904	-	-	-
IGF1 reduction, median, Q1; Q3 (%)	45 (19.5; 60.4)	35.7 (9.7; 59.8)	60.8 (41.2; 69)	0.158	-	-	-
Normalized IGF1 (n; %)	5; 35.7%	4; 23.5%	10; 66.7%	**0.041**	0.693^a^	0.031^a^	0.143^a^
Control of GH <2.5 μg/l (n; %)	6; 46.2%	9; 52.9%	8; 53.3%	0.841	-	-	-
Full biochemical control (n; %)	3; 23.1%	4; 23.5%	7; 46.7%	0.281	-	-	-
GM-SIR							
GH reduction, median, Q1; Q3 (%)	76.8 (48.7; 94.2)	54.2 (9.6; 85.2)	69.7 (31.3; 93.9)	0.333	-	-	-
IGF1 reduction, mean, SD (%)	39.1; 27.1	38.03; 28.92	52.49; 21.56	0.3	–	–	–
Normalized IGF1 (n; %)	2; 20%	10; 41.7%	7; 58.3%	0.191	-	-	-
Control of GH <2.5 μg/l (n; %)	6; 66.7%	9; 37.5%	8; 66.7%	0.173	-	-	-
Full biochemical control (n; %)	1; 11.1%	8; 33.3%	5; 41.7%	0.307	-	-	-
Three Tissue Method							
GH reduction, median, Q1; Q3 (%)	73.4 (26; 88.8)	54.8 (3.1; 88.2)	69.4 (60.2; 86.5)	0.747	-	-	**-**
IGF1 reduction, median, Q1; Q3 (%)	49.2 (28.7; 62.4)	44 (10.3; 63.5)	57.5 (37.6; 72.1)	0.488	-	-	**-**
Normalization of IGF1 (n; %)	7; 33.3%	7; 41.2%	5; 62.5%	0.362	-	-	**-**
Control of GH <2.5 μg/l (n; %)	10; 50%	8; 47.1%	5; 62.5%	0.765	-	-	-
Full biochemical control (n; %)	5; 25%	6; 35.3%	3; 37.5%	0.726	-	-	**-**

Bold values are statistically significant (p < 0.05).

a Bonferroni adjustment of p value necessary for multiple comparisons: statistical significance for p<0.0083.

HYPER, hyperintense; ISO, isointense; HYPO, hypointense somatotroph Pituitary Neuroendocrine Tumor; GM-SIR, gray matter signal intensity ratio; GH, growth hormone.

Univariate logistic regression showed significant associations only for the Visual Method: patients with ISO had a lower chance of achieving IGF1 normalization than patients with HYPO (odds ratio (OR) 0.089, confidence interval (CI) 0.015-0.538, p= 0.008), while such a tendency was not found between HYPER and HYPO (p=0.196).

### 3.6 GM-SIR as a quantitative approach to SI assessment

The GM-SIR (median, Q1;Q3) of the included patients was 0.90 (0.78; 1.12). It did not correlate with the age at diagnosis or at the onset, GH fasting and nadir concentrations, IGF1/ULN, GH/TV, post-treatment IGF1, or GH reduction. GM-SIR was weakly correlated with TV (R=0.248, p=0.041). We did not find any associations between GM-SIR and the frequency of GH control (<2.5 μg/l) or the frequency of the full biochemical control. GM-SIR could not predict the granulation pattern of the sPitNET in univariate models. ROC analysis was performed for GM-SIR to assess its potential ability to predict the IGF1 normalization after SSA, with a cutoff point of 1.13. However, the model was deemed statistically insignificant (area under the curve of 0.604, p=0.225).

### 3.7 Histopathology

In total, 31.7% of tumors expressed only GH and 17.1% co-expressed GH and PRL, while the remaining tumors showed GH and at least one other than PRL positive staining, among which the most frequent combination was GH, PRL, and αSU. We did not find differences between HYPER, ISO, and HYPO in immunophenotype, regardless of the SI assessment method. Furthermore, 58.3% of tumors were classified as densely granulated, 27.8% as sparsely granulated, and 13.9% as bihormonal sPitNETs in electron microscopy. According to the Visual Method and GM-SIR, HYPER and ISO were classified in electron microscopy as both densely and sparsely granulated. Bihormonal tumors presented only as HYPO (60-80%) and ISO (20- 40%). According to the Three Tissue Method, no HYPO was classified as having sparse granulation. Detailed results of the histopathological verification of HYPER, ISO, and HYPO tumors are presented in **Table 7**. Ki-67 <1% was the most frequent finding among all tumor types in the 38 available cases. No HYPO had a high proliferative index of >3%. We found no significant differences in Ki-67 between SI groups according to all assessment methods.

**Table 7:** Results of electron microscope verification of the hyperintense, isointense, and hypointense somatotroph pituitary neuroendocrine tumors.

Tumor type	Densely granulated	Sparsely granulated	Bihormonal	
Visual Method				
HYPER (n=11)	72.7%	27.3%	0%	p=0.159
ISO (n=12)	50%	41.7%	8.3%	
HYPO (n=13)	53.8%	15.4%	30.8%	
GM-SIR				
HYPER (n=7)	71.4%	28.6%	0%	p=0.514
ISO (n=17)	52.9%	35.3%	11.8%	
HYPO (n=12)	58.3%	16.7%	25%	
Three Tissue Method				
HYPER (n=17)	64.7%	29.4%	5.9%	p=0.056
ISO (n=13)	53.8%	38.5%	7.7%	
HYPO (n=6)	50%	0%	50%	

HYPER, hyperintense; ISO, isointense; HYPO, hypointense somatotroph Pituitary Neuroendocrine Tumor; GM-SIR gray matter signal intensity ratio.

## 4 Discussion

The unique radiological features of sPitNETs first drew attention in 2003 (4). The majority of pituitary tumors appearing as hypointense in T2-weighted MR images are verified as somatotropinomas, and hypointensity was discovered almost exclusively in densely granulated sPitNETs. Since then, SI assessment and its associations with granulation pattern (4, 6, 10), response to SSA (6, 7, 10), and pasireotide (9) have been studied. SI has been recommended as a tool in the treatment decision process by the recent guidelines on acromegaly (1, 2, 11). However, no consensus on SI assessment methods has been reached, as presented in **Table 1**. In our study, compatibility of the SI assessment methods ranged between 58% and 75.4%. Each method provided different proportions of tumors assigned to SI categories. As shown in **Table 2** and **Supplementary Table 1**, ISO represented 39% to 49.3% of the analyzed sPitNETs, forming a significant portion of the entire group according to all of the SI assessment methods. However, our data indicate the need for a unified definition of isointensity. Our observed frequency of HYPO (18.8% to 34.7%) aligns with the range reported in previous studies (**Table 1**). We noted a tendency for a higher frequency of HYPER using the Three Tissue Method (40.6% vs. 26.1% for the Visual Method and 18.8% for GM-SIR). Additionally, the Three Tissue Method tended to classify as HYPO less often (18.8% vs. 34.7% for the Visual Method and 31.9% for GM-SIR), consistent with existing data (12). Previously, Potorac et al. undertook efforts to confirm the unanimity of the qualitative and quantitative methods. They verified 29 visually assessed cases using ROI-based SI measurement, reaching 93% compatible results (14). However, differently from our study, the reference structures included healthy pituitary tissue and temporal grey matter.

With the Three Tissue Method, patients with ISO were younger at diagnosis and at the onset of symptoms than patients with HYPO. Differences in age have not been reported between patients with various tumor intensities (5, 7, 14). GM-SIR-based division revealed a high frequency of females among patients with HYPER (86%), consistent with already published data (7). Potorac et al. reported HYPER and ISO to be less biochemically active than HYPO (7, 14), while another study on 45 patients with acromegaly showed that HYPER differed significantly in terms of GH and IGF1/ULN from ISO and HYPO (5). In our study, GH/TV tended to be higher in HYPO than in ISO and HYPER, with no differences between HYPER and ISO. Hyperintensity has been associated with a lower biochemical activity relative to TV (5). We observed a tendency that both HYPER and ISO reached a larger TV than HYPO, again, with no significant difference between HYPER and ISO. Similar tendencies were presented in a subset of macroadenomas (12), while in other studies, ISO has been reported to be even smaller than HYPO (5) or to behave less invasively than HYPER (13). Our results indicate clinical similarities between HYPER and ISO. Further multi-center studies should investigate these aspects of ISO to clarify whether they constitute a separate clinical entity or should be interpreted together with HYPER.

In terms of response to preoperative SSA, we found differences in the frequency of IGF1 normalization only for the Visual Method, the univariate logistic regression confirmed its statistical significance in the prediction of IGF1 normalization. We did not observe differences in the frequency of full biochemical control between SI groups. Our results are partially supported by those previously published (12): SI has not been associated with overall biochemical control. However, for IGF1 normalization as a separate endpoint, only borderline associations were detected with SI assessed according to the GM-SIR and the Three Tissue Method. Univariate associations between SI and GH control <2.5 μg/l for the Visual Method and GM-SIR were also documented with statistical significance proven only between HYPO and ISO. However, only patients with macroadenomas were included in this study (12) and they received presurgical pharmacological treatment for a period of 48 weeks in comparison to 3-6 months in our study and other studies (5, 6, 15). In other articles, the intensity of sPitNETs could not differentiate between responders and non-responders in terms of IGF1, even though such an association has been confirmed for GH alone and differences in GH and IGF1 reduction have been observed between HYPER, ISO, and HYPO (5, 6).

In our patients, HYPER and ISO were classified as both densely and sparsely granulated. In the literature, the Visual Method has been associated with the granulation pattern of sPitNETs (19). According to our Three Tissue Method, no HYPO had sparse granulation, a finding that has already been observed with other SI assessment methods (5, 6). The GM-SIR did not predict the sPitNET’s granulation pattern in our study, contrarily to previous research that established associations between qualitative and quantitative SI assessment methods and the granulation type (19). For the purpose of our analyses, we separated a group of bihormonal tumors, constituting a significant percentage of sPitNETs, following Varlamov et al. Interestingly, our bihormonal tumors presented only as HYPO (60-80%) and ISO (20- 40%), which has not been reported before (20).

A strength of our study is the number of patients, which is considerable for a single-center observation, and includes, unlike other reports (9, 12), consecutive, unselected patients with acromegaly. To our knowledge, ours is the largest group of unselected patients with acromegaly, in which all these methods were compared. We used widely available radiological software; quantification was quick, easy, and applicable for external MRI. The use of unselected MR images provides a good generalization of the results. We used strict criteria of biochemical response (full biochemical control, IGF1/ULN<1, GH concentration <2.5 μg/l), and patients were pretreated with SSA for a limited period of 3-6 months. This provides a subset of patients who respond to SSA well and quickly. The limitations of this study include the fact that this is a single-center observation. Data collection was partially retrospective, hence the missing MRI and histopathology, resulting in a significant number of patients being excluded from the analyses.

## 5 Conclusions

Our study compared different qualitative and quantitative methods of assessment of the T2-weighted SI of sPitNETs. ISO is the dominating SI group according to all the methods we used. They present radiological and biochemical features similar to HYPER. Whether ISO should be considered a separate SI group or constitute a single entity together with HYPER requires further research. Out of the 3 compared methods, the SI groups according to the Visual Method better correlated with IGF1 control after SSA treatment. Further multi-center studies are required to unify the SI assessment and to prove its applicability in the everyday management of acromegaly.

## Data Availability

The original contributions presented in the study are included in the article/**Supplementary Material**. Further inquiries can be directed to the corresponding author.

## References

[1] Katznelson L Laws ER Melmed S Molitch ME Murad MH Utz A. Acromegaly: an endocrine society clinical practice guideline. J Clin Endocrinol Metab. (2014) 99:3933–51. doi: 10.1210/jc.2014-2700 25356808

[2] Fleseriu M Biller BMK Freda PU Gadelha MR Giustina A Katznelson L. A Pituitary Society update to acromegaly management guidelines. Pituitary. (2021) 24:1–13. doi: 10.1007/s11102-020-01091-7 33079318 PMC7864830

[3] Bolanowski M Ruchała M Zgliczyński W Kos-Kudła B Hubalewska-Dydejczyk A Lewiński A. Diagnostics and treatment of acromegaly — updated recommendations of the Polish Society of Endocrinology. Endokrynol Pol. (2019) 70:2–10. doi: 10.5603/EP.a2018.0093 30843181

[4] Hagiwara A Inoue Y Wakasa K Haba T Tashiro T Miyamoto T. Comparison of growth hormone-producing and non-growth hormone-producing pituitary adenomas: Imaging characteristics and pathologic correlation. Radiology. (2003) 228:533–8. doi: 10.1148/radiol.2282020695 12819334

[5] Heck A Ringstad G Fougner SL Casar-Borota O Nome T Ramm-Pettersen J. Intensity of pituitary adenoma on T2-weighted magnetic resonance imaging predicts the response to octreotide treatment in newly diagnosed acromegaly. Clin Endocrinol (Oxf). (2012) 77:72–8. doi: 10.1111/j.1365-2265.2011.04286.x 22066905

[6] Heck A Emblem KE Casar-Borota O Bollerslev J Ringstad G. Quantitative analyses of T2-weighted MRI as a potential marker for response to somatostatin analogs in newly diagnosed acromegaly. Endocrine. (2015) 52:333–43. doi: 10.1007/s12020-015-0766-8 26475495

[7] Potorac I Petrossians P Daly AF Alexopoulou O Borot S Sahnoun-Fathallah M. T2-weighted MRI signal predicts hormone and tumor responses to somatostatin analogs in acromegaly. Endocr Relat Cancer. (2016) 23:871–81. doi: 10.1530/ERC-16-0356 27649724

[8] Puig-Domingo M Resmini E Gomez-Anson B Nicolau J Mora M Palomera E. Magnetic resonance imaging as a predictor of response to somatostatin analogs in acromegaly after surgical failure. J Clin Endocrinol Metab. (2010) 95:4973–8. doi: 10.1210/jc.2010-0573 20739382

[9] Coopmans EC Schneiders JJ El-Sayed N Erler NS Hofland LJ van der Lely AJ. T2-signal intensity, SSTR expression, and somatostatin analogs efficacy predict response to pasireotide in acromegaly. Eur J Endocrinol. (2020) 182:595–605. doi: 10.1530/EJE-19-0840 32375119

[10] Dogansen SC Yalin GY Tanrikulu S Tekin S Nizam N Bilgic B. Clinicopathological significance of baseline T2-weighted signal intensity in functional pituitary adenomas. Pituitary. (2018) 21:347–54. doi: 10.1007/s11102-018-0877-3 29460202

[11] Puig-Domingo M Bernabéu I Picó A Biagetti B Gil J Alvarez-Escolá C. Pasireotide in the personalized treatment of acromegaly. Front Endocrinol (Lausanne). (2021) 12:648411. doi: 10.3389/fendo.2021.648411 33796079 PMC8008639

[12] Bonneville F Rivière LD Petersenn S Bevan JS Houchard A Sert C. MRI T2 signal intensity and tumor response in patients with GH-secreting pituitary macroadenoma: PRIMARYS *post hoc* analysis. Eur J Endocrinol. (2019) 180:155–64. doi: 10.1530/EJE-18-0254 30540560

[13] Alhambra-Expósito MR Ibáñez-Costa A Moreno-Moreno P Rivero-Cortés E Vázquez-Borrego MC Blanco-Acevedo C. Association between radiological parameters and clinical and molecular characteristics in human somatotropinomas. Sci Rep. (2018) 8:6173. doi: 10.1038/s41598-018-24260-y PMC5906631 29670116

[14] Potorac I Petrossians P Daly AF Schillo F Ben SC Nagi S. Pituitary MRI characteristics in 297 acromegaly patients based on T2-weighted sequences. Endocr Relat Cancer. (2015) 22:169–77. doi: 10.1530/ERC-14-0305 25556181

[15] Shen M Zhang Q Liu W Wang M Zhu J Ma Z. Predictive value of T2 relative signal intensity for response to somatostatin analogs in newly diagnosed acromegaly. Neuroradiology. (2016) 58:1057–65. doi: 10.1007/s00234-016-1728-4 27516099

[16] Lewis D Roncaroli F Kearney T Coope DJ Gnanalingham K. Quantitative magnetic resonance-derived biomarkers as predictors of function and histotype in adenohypophyseal tumours. Neuroendocrinology. (2022) 112:276–86. doi: 10.1159/000516823 33902055

[17] Bogusławska A Gilis-Januszewska A Godlewska M Nowak A Starzyk J Hubalewska-Dydejczyk A. Gender and age differences among patients with acromegaly. Pol Arch Intern Med. (2022) 132:16232. doi: 10.20452/pamw.16232 35289160

[18] Petersenn S Houchard A Sert C Caron PJ. Predictive factors for responses to primary medical treatment with lanreotide autogel 120 mg in acromegaly: *post hoc* analyses from the PRIMARYS study. Pituitary. (2020) 23:171–81. doi: 10.1007/s11102-019-01020-3 PMC7066297 31879842

[19] Park YW Kang Y Ahn SS Ku CR Kim EH Kim SH. Radiomics model predicts granulation pattern in growth hormone-secreting pituitary adenomas. Pituitary. (2020) 23:691–700. doi: 10.1007/s11102-020-01077-5 32851505

[20] Varlamov EV Wood MD Netto JP Thiessen J Kim J Lim DST. Cystic appearance on magnetic resonance imaging in bihormonal growth hormone and prolactin tumors in acromegaly. Pituitary. (2020) 23:672–80. doi: 10.1007/s11102-020-01075-7 32870441

