# Supplementary material for: Quantitative and qualitative assessment of a pituitary neuroendocrine tumor’s T2-signal intensity in acromegaly – a call for unification
Source: Front Endocrinol (Lausanne). 2024 Nov 20;15:1441745. doi: 10.3389/fendo.2024.1441745 (PMC11618535; doi:10.3389/fendo.2024.1441745)
Supplement: Supplementary file 1 [file Table1.pdf]

Supplementary Table 1. Comparison of Signal Intensity classification of the Somatotroph Pituitary Neuroendocrine Tumors according to various methods of Signal Intensity assessment

Comparison of Signal Intensity classification of the sPitNETs according to various methods of Signal Intensity assessment					
Visual Method vs. GM-SIR		GM-SIR<br>Frequency of the sPitNET type according to signal intensity (n)			
		HYPER	ISO	HYPO	p value
Visual Method Frequency of the sPitNET type according to signal intensity (n)	HYPER	11	7	0	**<0.001**
	ISO	2	22	3	
	HYPO	0	5	19	
Visual Method vs. Three Tissue Method		Three Tissue Method<br>Frequency of the sPitNET type according to signal intensity (n)			
		HYPER	ISO	HYPO	p value
Visual Method Frequency of the sPitNET type according to signal intensity (n)	HYPER	16	2	0	**<0.001**
	ISO	12	13	2	
	HYPO	0	13	11	
GM-SIR vs. Three Tissue Method		Three Tissue Method<br><br>Frequency of the sPitNET type according to signal intensity (n)			
		HYPER	ISO	HYPO	p value
GM-SIR<br><br>Frequency of the sPitNET type according to	HYPER	13	0	0	**<0.001**
	ISO	15	19	0	
	HYPO	0	9	13	

signal intensity (n)					

Bold values are statistically significant ($p < 0.05$).

Abbreviations: sPitNET- somatotroph Pituitary Neuroendocrine Tumor, HYPER- hyperintense, ISO- isointense, HYPO- hypointense somatotroph Pituitary Neuroendocrine Tumor, GM-SIR gray matter signal intensity ratio
